# Pioneering family medicine: A collaborative global health education partnership in Ethiopia

**DOI:** 10.4102/phcfm.v16i1.4599

**Published:** 2024-09-24

**Authors:** Meseret Z. Woldeyes, Leila Makhani, Nitsuh Ephrem, Jamie Rodas, Ellena Andoniou, Katherine Rouleau, Abbas Ghavam-Rassoul, Praseedha Janakiram

**Affiliations:** 1 Department of Family Medicine, Addis Ababa University, Addis Ababa, Ethiopia; 2 Department of Family and Community Medicine, Temerty Faculty of Medicine, University of Toronto, Toronto, Canada; 3 Wilson Centre, University Health Network, Toronto, Canada; 4 The Institute for Education Research, University Health Network, Toronto, Canada; 5 Department of Family and Community Medicine, Unity Health, Toronto, Canada

**Keywords:** medical education, residency programme, family medicine, Ethiopia, TAAAC-FM, global health partnership

## Abstract

In 2013, Ethiopia launched its first Family Medicine (FM) residency programme at Addis Ababa University (AAU). The University of Toronto’s Department of Family and Community Medicine (DFCM) was invited to support Addis Ababa University’s Department of Family Medicine’s (AAU-FM) educational programme activities forming the Toronto Addis Ababa Academic Collaboration in Family Medicine (TAAAC-FM). This paper describes the TAAAC-FM partnership, a capacity-strengthening initiative that focuses on four key levers of academic engagement and transformation: education offerings for AAU-FM trainees, partnership preparation of DFCM faculty, fostering AAU-FM faculty development and leadership, and lastly scholarship, knowledge sharing and mentorship. Toronto Addis Ababa Academic Collaboration in Family Medicine operates on principles of respect, flexibility and cultural sensitivity. Monthly virtual meetings and annual in-person faculty visits fostered curriculum support, teaching and leadership training, ensuring that the programme remained responsive to evolving needs. The partnership has contributed to a Community of Practice (CoP) to advance FM in Ethiopia, promoting shared learning. Addis Ababa University’s Department of Family Medicine faculty leads in various roles, engages with global FM communities, and contributes to policy development, demonstrating significant progress in FM education and leadership. Looking ahead, TAAAC-FM aims to adapt its efforts based on the capacity built with AAU-FM, continue faculty development, and strengthen linkages within the global healthcare community. The partnership’s success underscores the importance of collaborative, culturally informed high-low resource setting approaches to FM training and healthcare system strengthening, offering valuable insights for similar initiatives.

## Introduction

In 2013, in line with a wave of similar efforts across the African region, Ethiopia launched its first Family Medicine (FM) programme at Addis Ababa University, College of Health Sciences, School of Medicine.^1,2^ The Toronto Addis Ababa Academic Collaboration in Family Medicine (TAAAC-FM) is a capacity-strengthening partnership established between Addis Ababa University’s Department of Family Medicine (AAU-FM) and the Department of Family and Community Medicine (DFCM) at the University of Toronto (U of T) to support Ethiopia’s inaugural FM residency programme.^3^ The TAAAC-FM collaboration was built on a pre-existing model initially established between the departments of psychiatry at AAU and U of T. One of the distinctive features of the model is the presence of two U of T faculty and one senior learner in Addis for 1 month, three times per year.

This paper describes this unique institutional collaboration and identifies four key levers of academic engagement and partnership that contributed to the ongoing evolution of Ethiopian family medicine. Lastly, it distils some of the main lessons learned to date.

## Setting the socio-political scene: Establishing family medicine in Ethiopia

Primary health care (PHC), as a whole-of-system approach to health and wellbeing, has been recognised by the World Health Organization (WHO) as the necessary pathway to achieve universal health coverage (UHC) and other health related Sustainable Development Goals (SDGs). It includes as one of three essential components, primary care and essential public health functions at the core of integrated health services. Family medicine is a medical discipline that inherently drives toward high-quality primary care. Primary care, as a core component of PHC, delivers first-contact, accessible, continued, comprehensive, coordinated and person-focused care. Primary care is pivotal in bolstering PHC-oriented systems and advancing UHC through person-centred care across the lifespan.^4^

In 1991, in an effort to address one of the most severe shortages of physicians in the world^5^ and orient towards a PHC approach,^6^ the Ethiopian Federal Ministry of Health launched ambitious Health Sector Development Plans (1991–2015). Significant emphasis was placed on developing human resources for health (HRH) including increasing the number of physicians and enhancing postgraduate training programmes.^7^ The establishment of a FM postgraduate training programme in Ethiopia emerged in response to the prioritisation of the development of HRH and in particular of comprehensive, patient-centred, community-oriented primary care.

Philpott et al. detail the conceptualisation, early development, and launch of the country’s first-ever AAU-FM residency in Ethiopia in 2013.^1^ The institutional partnership now known as the TAAAC-FM is an invited partnership from the emerging AAU-FM department to share the experience and expertise of Canadian family medicine, expecting that it would be reshaped, adapted, and contextualised to meet the specific needs of Ethiopia.

## Programme overview

Since 2013, over 40 DFCM faculty, 10 current AAU faculty, and over 70 graduated residents from AAU-FM have participated in 44 virtual teaching sessions, and 31 on-site teaching trips supported with in-kind contributions from both AAU and DFCM. The TAAAC-FM collaboration is grounded in shared contributions from both partners and has been supported by transformative gifts and in-kind supports, which have exponentially grown the capacity of this partnership over the past 10 years. It is grounded in deep commitment, dedication, and trust. Table 1 provides a snapshot of the key programme features.

**TABLE 1:** Addis Ababa University (AAU) Family Residency Programme snapshot.

Key features of the programme	Details of each programme feature
Length of training	3.5-year family medicine residency programme
Qualification conferred	Postgraduate specialty certificate in family medicine
Internship site rotations	1 Referral hospital 1 Secondary hospital 1 Community Health Centre* 1 Community-based Organisation (CBO) delivering healthcare services*
Continuous programmatic assessment	Formative and Summative (e.g., site or attachment evaluation, portfolio assessment, academic half day presentations and quizzes, written examinations, Objective Structured Clinical Exam [OSCE], progressive evaluation, research and quality improvement projects)

* , In addition to in-class FM training at AAU, residents participate in a year-long clinical attachment at an affiliated community health centre and CBO, as part of the 3.5-year programme. The internship includes half-days in Family Medicine, Paediatrics, Obstetrics/Gynaecology, Internal Medicine, General Surgery, Emergency Medicine, Anaesthesia, Psychiatry, Dermatology, etc., and focuses on community-based family medicine training.

## Responsive innovations: Bespoke Toronto Addis Ababa Academic Collaboration in Family Medicine elements that transcend boundaries

A first-of-its-kind programme in Ethiopia, TAAAC-FM is guided by principles of collaboration, respect, sustainability, responsiveness, and flexibility. Collaborative activities focus on FM’s scholarly foundations. These included curriculum development and adaptation, learner evaluation, teaching (didactic, case-based, and skills-based training), faculty and leadership development, and scholarship or knowledge sharing as a form of advocacy (Table 2).

**TABLE 2:** TAAAC-FM Model of 4 key levers of academic engagement and transformation by DFCM faculty.

Area	Examples of academic activities
Education offerings for AAU-FM trainees	1 month in-person teaching trips: 2 DFCM faculty, 1 senior learner Academic half-day teaching Clinical bedside supervision of learners Exam assessment preparatory consultation and/or advisory roles Invited external examiner roles Procedural skills-based workshops Covid response: Virtual asynchronous co-teaching modules Covid response: Virtual asynchronous module quizzes
Partnership orientation and preparation of DFCM faculty	Pre-departure orientation phase 1 Pre-departure orientation phase 2 Remote support of DFCM faculty and learners while on teaching trip Post-return debrief for travelling faculty and learners Orientation for new interested DFCM faculty Building a global health community within DFCM
Fostering AAU-FM faculty development and leadership	In person faculty development sessions at AAU-FM Covid response: virtual synchronous faculty development, as needed Mentorship in leadership and programme initiatives co-development: Monthly phone and/or Zoom meetings Leadership advancement: In-person and/or virtual attendance of AAU-FM faculty at Toronto International Programme to Strengthen Primary Care Leadership advancement: Virtual, Advancing Women’s Excellence in Family Medicine: A programme for Emerging Leaders, DFCM Co-Attendance at the College of Family Physicians of Canada Family Medicine Forum, Besrour Centre for Global Family Medicine, World Organization of Family Doctors (WONCA) events, and the Primary Care and Family Medicine (Primafamed) network in sub-Saharan Africa Sponsorship of networking opportunities with new GH partnerships
Scholarship, knowledge sharing and mentorship	1st year residents’ quality improvement projects, when invited for co-consultation 3rd year residents’ research projects, invited external examiner role Faculty research mentorship, when requested Sponsorship for conference attendance Co-presentation at conferences Co-submission of posters and/or oral presentations and/or manuscripts and/or Research Assistant support

AAU-FM, Addis Ababa University’s Department of Family Medicine; DFCM, Department of Family and Community Medicine; COVID-19, coronavirus disease 2019; TAAAC-FM, Toronto Addis Ababa Academic Collaboration in Family Medicine.

Teaching activities delivered during the month-long DFCM faculty visits or virtually are jointly planned by AAU-FM and DFCM faculty using a needs-based approach. This supports strategic decision-making and resource allocation that prioritises the evolving needs and preferences of the programme as identified by the AAU-FM programme director, leadership and faculty.

Monthly virtual meetings bring together TAAAC-FM leaders from AAU and the DFCM to debrief, discuss, plan, and problem-solve in ways that are respectful of culture, academic context, and values. In-person requested teaching, during the month-long teaching trips, is delivered jointly by DFCM and AAU-FM faculty at academic teaching days, community health centres, and on the wards. Support for the examination and certification of trainees has included exam development and review with DFCM examiners on invitation by the AAU-FM leadership. The coronavirus disease 2019 (COVID-19) pandemic necessitated a pivot that included the introduction of virtual co-teaching, and virtual faculty development, all of which are now integrated into the collaborative teaching programme as needed.

To facilitate and orient DFCM faculty to their roles within the TAAAC-FM collaboration, the DFCM provides detailed FM-specific orientation to the principles of TAAAC-FM alongside consultation with AAU-FM faculty to inform teaching priorities and preparation (Table 2). Faculty development and leadership development has been a priority focus for TAAAC-FM, with virtual and in-person sessions at AAU-FM, as well as leadership course offerings at U of T focused on capacity-building for FM leaders (Table 2). Addis Ababa University’s Department of Family Medicine faculty have been sponsored to present scholarly work and attend key family medicine conferences as a mechanism to enhance networking and building a FM community.^8^

## Partnership, growth, and reaching beyond Toronto Addis Ababa Academic Collaboration in Family Medicine

The TAAAC-FM collaboration is committed to the principles of allyship, including a shared vision and goals, mutual trust and respect, a bidirectional flow of knowledge, flexibility, and navigating challenges together. These principles, further outlined in Table 3, are critical to the success of this partnership. Addis Ababa University’s Department of Family Medicine delivers a robust 3.5-year residency programme, with its own autonomous leadership and oversight of the residency programme, operations, curriculum, and functions, while navigating the complexity of a newly emerging specialty in the healthcare system.^8^ Toronto Addis Ababa Academic Collaboration in Family Medicine has emphasised building trustful, relational leadership and communication, underpinning the institutional and country-context, to bolster AAU-FM’s growth and evolution.

**TABLE 3:** Main lessons learned and signposts for developing educational partnerships in family medicine.

Lessons learned and signposts
**Shared vision and goals:** Aligning focused, constructive partnership support on AAU-FM academic priorities, goals, values, and strategies. Such alignment enhances the effectiveness and impact of the collaboration.
**Commitment and trust:** A strong partnership thrives on commitment, dedication, and trust among all parties. Consistent engagement and open communication help to build and maintain trust, which is foundational for long-term success.
**Respect for cultural context:** Respecting the history, culture, and values of each partner country (Ethiopia & Canada, in this case) fosters a respectful and inclusive environment. These efforts are continuous, building on the infrastructure, guidance and leadership of the past 10 years.
**Sustainable capacity strengthening:** Focus on strengthening sustainable capacity that leads to the expansion of programmes and enhancement of skills. Developing local expertise ensures that the benefits of the partnerships continue to grow and adapt beyond the initial phases of the collaboration.
**Bidirectional knowledge flow:** Promote a two-way exchange of knowledge and experiences. This bidirectional flow enriches both partners and contributes to mutual learning and development.
**Responsiveness and flexibility:** Be responsive and flexible to adapting and customising programmes according to the changing needs and contexts of the partners. Adaptability is essential for addressing emerging challenges and opportunities effectively.
**Persistence in adversity:** Embrace persistence and perseverance in overcoming challenges and difficult situations. Navigating complexities and setbacks with resilience contributes to building a partnership that can endure and succeed over time.

AAU-FM, Addis Ababa University’s Department of Family Medicine.

Both DFCM and AAU-FM faculty and learners have learned to recognise and address the operational challenges of communication, co-design of contextually relevant teaching materials, and participating in orienting and debriefing activities. Department of Family and Community Medicine teaching faculty are oriented to the scope of the role as a partner, valuing skills of remaining flexible to changing programme circumstances, upholding AAU-FM’s identified academic expectations, adapting to identified curriculum gaps and learning needs, while teaching in areas of strength for all partners.

Addis Ababa University’s Department of Family Medicine faculty are new to the FM landscape but have leveraged the TAAAC-FM partnership to lead and represent the specialty while engaging multiple stakeholders. Addis Ababa University’s Department of Family Medicine faculty are increasingly engaged in FM associations and stakeholder organisations both within Ethiopia, and beyond. Addis Ababa University’s Department of Family Medicine faculty and graduates hold leadership positions at AAU, serving as Department Heads and committee leads. They are founding members of the Ethiopian Society of Family Physicians who actively engage with the Ministry of Health to shape policy and curriculum and collaborate with non-governmental organisations, including Hospice Ethiopia. Addis Ababa University’s Department of Family Medicine faculty are participating in global family medicine communities including the Canadian College of Family Physicians, the Besrour Centre for Global Family Medicine in Canada, the Primary Care and Family Medicine (PRIMAFAMED) network in sub-Saharan Africa, and the World Organization of Family Doctors. These intentional efforts strengthen connections to broader FM organisations, supporting the development of academic leaders, advocates, and scholars at both AAU-FM and the DFCM.

## Establishing a community of practice: Through transformative collaboration and education

The Toronto Addis Ababa Academic Collaboration in Family Medicine is cultivating a Community of Practice (CoP) as a group that is connected together through shared vision, knowledge, and interest,^9^ to support an emerging FM community that promotes joint learning to improve healthcare service, practice, and delivery. The Toronto Addis Ababa Academic Collaboration in Family Medicine has placed value on forging strong FM faculty connections that extend beyond AAU-FM. Shared resources, sharing lived experience as practising family doctors and educators, co-teaching at AAU, co-presenting, and co-designing new initiatives have been tangible CoP building opportunities for both AAU-FM and DFCM faculty. Addis Ababa University’s Department of Family Medicine and DFCM continue to build this CoP to address shared practice interests, knowledge, and concerns.

Furthermore, DFCM faculty work alongside AAU-FM faculty during clinical supervision of learners and didactic teaching when on site. This further fosters reciprocal learning experiences for both AAU-FM and DFCM faculty on themes of clinical presentations, a culture of feedback and preceptorship, cultural humility, and interactive teaching styles.

## Reflection: Distilling the main lessons learned from the Toronto Addis Ababa Academic Collaboration in Family Medicine collaborative partnership

Strengthening FM has been linked to better health outcomes, lower costs, and improved health equity.^10^ With comprehensive training and resources, FM specialists can significantly contribute to patient care in the community, health centres, clinics, and hospitals, thereby reshaping the healthcare landscape towards PHC.^2^ The internationalisation of medical education through TAAAC-FM comes with a recognition that all offerings evolve based on the AAU-FM specific needs and capacity identified. The offerings are dynamic rather than standardised, and teaching themes or skills are adapted to address the gaps identified by AAU-FM faculty, recognising AAU-FM growth and internal capacity annually. The next phase of the TAAAC-FM partnership aims to adapt collaborative efforts through consideration of AAU-FM’s strength and capacity, principles of allyship, recognition of AAU-FM’s request for ongoing faculty development, while fostering linkages between the Ethiopian and global healthcare community. Professional development will continue to be scaffolded to meet needs identified by AAU faculty while strengthening the knowledge pool of faculty partners at both institutions. The challenges of the COVID-19 pandemic and human resource limitations in FM in both institutional climates are critical considerations going forward. Bi-directional learning in particular has transformed the lens of partnership work for the DFCM, including emphasis on active listening to evolving AAU priorities, strengthening DFCM’s contextual humility, development of orientation and debrief processes, clinical learning while reflecting on health workforce challenges, and considerations for models of care in the future. The DFCM and AAU have learned many lessons in the development of this educational partnership and some of the main lessons to date are shared in Table 3.

## Conclusion

This report provides an overview crafted by the past FM leadership of experiences and insights gained over the 10 years. The innovative TAAAC-FM partnership has highlighted four key levers in high-low resource setting academic engagement and transformation: strengthening education, orientation through preparatory contextualisation, faculty development and leadership, and knowledge dissemination or scholarship. Through these levers, TAAAC-FM has striven to: (1) strengthen teaching capacity in FM for the enhanced delivery of primary care; (2) establish a knowledge-sharing CoP that encourages collaboration and partnership; and (3) enhance local-to-global leadership opportunities with rich lessons learned. These insights may have value to similar partnerships, and highlight the rich expertise, wisdom, collaboration, and friendship of this impactful allyship between the AAU-FM and the DFCM in their joint commitment to advancing primary care and health system strengthening.
